# Persistence and recurrence in tumor-induced osteomalacia: A systematic review of the literature and results from a national survey/case series

**DOI:** 10.1007/s12020-022-03039-2

**Published:** 2022-04-05

**Authors:** Luisella Cianferotti, Chiara Delli Poggi, Francesco Bertoldo, Carla Caffarelli, Chiara Crotti, Davide Gatti, Sandro Giannini, Stefano Gonnelli, Maurizio Mazzantini, Viapiana Ombretta, Stefania Sella, Angela Setti, Massimo Varenna, Francesca Zucchi, Maria Luisa Brandi

**Affiliations:** 1 Department of Experimental, Clinical and Biomedical Sciences, University of Florence, Bone Metabolic Diseases Unit, University Hospital of Florence, largo Palagi 1, 50139 Florence, Italy; 2 Department of Medicine, University of Verona, Piazzale LA Scuro 10, Policlinico GB Rossi, Piazzale LA Scuro 10, 37134 Verona, Italy; 3 Department of Medicine, Surgery and Neuroscience, University of Siena, Policlinico Le Scotte, Viale Bracci 2, 53100 Siena, Italy; 4 Bone Diseases Unit, Department of Rheumatology, Gaetano Pini Institute, Via Pini, 9, Milan, 20122 Italy; 5 Rheumatology Section, Department of Medicine, University of Verona Hospital Trust, Policlinico G.B. Rossi, Piazzale LA Scuro 10, 37134 Verona, Italy; 6 Department of Medicine (DIMED), Clinica Medica Uno, University of Padua, via Nicolò Giustiniani, 2, 35128 Padua, Italy; 7 Rheumatology Unit, and Fracture Liaison Service, University Hospital of Pisa, via Roma 67, 56126 Pisa, Italy; 8 FIRMO Foundation, via san Gallo 123, Florence, Italy

**Keywords:** TIO, Hypophosphatemia, FGF23, Burosumab, Phosphate

## Abstract

**Purpose:**

Tumor induced osteomalacia (TIO) is a rare disease of mineral metabolism, whose clinical picture is dominated by hypophosphatemia usually due to an excess of circulating FGF23 produced by small mesenchymal tumors. Data on the real prevalence of the disease are lacking, with the knowledge of the disease mainly relying on case reports and small case series. No estimate is available on the prevalence of uncured TIO.

**Methods:**

National multi-center, cross-sectional and retrospective study on persistent or recurrent cases of TIO followed in referral centers for bone diseases**;** systematic review of the published persistent and recurrent cases of TIO. Data from patients consecutively evaluated in referral Italian centers for bone diseases were collected; a PubMed search on persistent, recurrent and unoperable cases of TIO was carried out.

**Results:**

Sixteen patients (mean age at diagnosis 52.5 ± 10.6 years) with persistent (*n* = 6, 37,5%), recurrent (*n* = 7, 43.7%) or not operable (*n* = 3, 18.8%) TIO were described. Delay in diagnosis (2.5 ± 1.3 years) was demonstrated. All patients experienced fragility fractures or pseudofractures and disabling bone and muscle pain. BMD was significantly reduced (mean T-score −2.7 ± 1.7 and −2.7 ± 0.9 at lumbar spine and femoral neck, respectively). Fourteen patients were maintained under therapy with phosphate salts and calcitriol, while in 2 patients therapy with burosumab, an anti-FGF23 antibody, was commenced.

**Conclusion:**

A significant number of patients with TIO remain either undiagnosed for tumor localization or tumor recur or persist after surgery. These patients with active disease represent possible candidates for burosumab treatment.

## Introduction

Tumor-induced osteomalacia (TIO), also referred to as oncogenic osteomalacia (OO) or oncogenic hypophosphatemic osteomalacia (OHO), is a rare paraneoplastic syndrome caused by overproduction of fibroblast growth factor 23 (FGF-23) by small, usually benign, mesenchymal tumors originating in soft tissues or bone. A small number of TIO cases is also reported in association with neurofibromatosis and McCune-Albright syndrome, or with carcinomas (i.e., anaplastic thyroid carcinoma, breast carcinoma) [1, 2].

Less than 1000 cases of TIO are reported in the literature, and a vast majority of studies consists of case reports and small case series. Therefore, there is a lack of data on real prevalence and incidence of disease [3]. However, several studies suggest that tumor-induced osteomalacia is the most common acquired cause of FGF23-induced hypophosphatemia, has a medium onset age of 40–45 years, and affects both genders equally [4].

The first case was described in 1947 by McCance [5], but the link between tumors and oncogenic osteomalacia was established only in 1959 [6].

Any mesenchymal tumor can cause TIO, including hemangiopericytomas, various types of vascular neoplasms, giant cell-rich tumors of bone, osteosarcomas, enchondromas, osteoblastomas, and others [7]. Rich vascularization and high proliferation indexes are peculiar histological features [1].

TIO is characterized by progressive muscle weakness, bone pain, and fractures (especially in the ribs, vertebral bodies, and proximal femur). Clinical presentation is often dramatic: previously healthy individuals quickly develop profound disabilities [8]. The typical biochemical features of these patients are hypophosphatemia, due to renal phosphate wasting, and low or inappropriately normal 1,25 dihydroxy-vitamin D (1,25(OH)_2_D_3_), due to excessive secretion of tumor-derived FGF-23 [9–11].

In chronic hypophosphatemia, bone is characterized by typical signs of osteomalacia, with widened osteoid seams and delayed mineralization [12].

Since the symptoms are non-specific and phosphate levels are not routinely evaluated, patients are often misdiagnosed with a variety of rheumatological, musculoskeletal, or psychiatric disorders, and experience a progressive and significant deterioration of quality of life, with a medium delay in diagnosis from 2.5 to 28 years [13].

Moreover, once the disease is diagnosed, identification of the tumor (usually performed by anatomical and functional imaging, i.e., ^68^Ga-DOTA PET/CT, Technetium 99 m octreo-SPECT, OctreoScan SPECT/TC, ^18^FDG PET/TC) is often difficult since tumors are, in most cases, small, slow growing, and located in almost any part of the body [14].

Once the TIO-associated tumor is identified, the current standard of care is surgical resection with clear-margin, often with adjuvant radiotherapy. Biochemical abnormalities usually resolve in a few days after the intervention, followed by dramatic improvement in clinical symptoms within a few weeks. Alternative therapies for patients not suitable for surgery include image-guided ablation with cryoablation, radiofrequency ablation, or radiotherapy [15].

In some patients, local recurrence of primary tumor may occur at varying periods of time. Relapse of TIO is sometimes, but rarely, associated with metastasis of the primary tumor, with the most frequent site of secondary lesions being the lungs. In some cases, it is not possible to localize or completely resect the primary tumor, and the tumor-induced osteomalacia persists, and medical treatment with phosphate supplementation and active vitamin D metabolites (calcitriol or alpha-calcidiol) is maintained [16]. Long-term medical treatment can lead to hypercalciuria (and subsequently nephrocalcinosis, nephrolithiasis, and renal failure) and secondary hyperparathyroidism. In some patients, replacement therapy with phosphate salts is unable to relieve symptoms of TIO, with a progressive decline of quality of life and impaired musculoskeletal performance [17].

Some studies have suggested the use of somatostatin analog octreotide as an alternative therapeutic strategy in not operable/not localized TIO-associated tumors positive at OctreoScan, but lack of long-term efficacy emerges from a vast majority of case reports [18].

Recently, burosumab, a human monoclonal antibody against FGF23, has been approved for the therapy of X-linked hypophosphatemia in children and adults, the most common heritable, genetically-determined form of FGF23-related hypophosphatemic disease [19]. Preliminary results demonstrate a significant improvement in symptoms, biochemical parameters, quality of life, and musculoskeletal performance [20, 21].

The use of burosumab in other types of FGF23-related hypophosphatemic rickets, such as TIO, has been proposed and is currently under investigation [22–24]. At this time, burosumab is available for compassionate use in patients in which surgical treatment of primary tumor is not feasible nor curative.

The purpose of this study is to describe for the first time a national multi-center experience with recurrent or persistent cases of TIO, to estimate and evaluate patients eligible for burosumab treatment. Patients with unresectable or undetectable tumors are also included. A comprehensive and updated review of literature regarding cases with recurrence or persistence of TIO is conducted.

## Materials and methods

### Study group

Data related to patients diagnosed with persistent or recurrent TIO attending Italian endocrinological, internal medicine, and rheumatological referral centers were collected: 6 from Gaetano Pini Institute Milano (Rheumatology Unit), 3 from University Hospital of Florence (Bone Metabolic Diseases Unit), 2 from University Hospital of Padova (Internal Medicine), 2 from University Hospital of Verona (Internal Medicine), 1 from University Hospital of Verona (Rheumatology Unit), 1 from University Hospital of Pisa (Rheumatology Unit), 1 from University Hospital of Siena (Internal Medicine).

Data were collected in a database and analyzed at the University of Florence.

“Recurrence of TIO” was diagnosed in patients whose disease relapsed after at least 6 months of normophosphatemia after local surgical treatment. “Persistence of TIO” was diagnosed in patients in which hypophosphatemia and related symptoms persisted after surgery or who relapsed within six months after tumor resection. Data about patients with not operable/not localized tumors were also collected.

General data, past medical history, TIO-related symptoms, biochemical profile, imaging studies done to localize the tumor, treatments, histopathology when available, and outcomes of therapies at diagnosis and at every subsequent re-evaluation, were collected.

Bone mineral density was assessed by DXA (Hologic densitometers, QDR 4500 and Discovery); T-score and Z-score parameters were indicated, when available.

Data were expressed as mean ± SD.

The study was approved by the local ethical committee and the patients gave appropriate informed consent for data collection.

### Literature review

A PubMed search referred to all original articles and review papers published up to November 2020. The research was carried out by the University of Florence by matching the following terms: “Tumor induced osteomalacia AND recurrence”, “Tumor induced osteomalacia AND recurrent”, “Tumor induced osteomalacia AND persistence”, “Tumor induced osteomalacia AND persistent”, “TIO AND recurrence”, “TIO AND recurrent”, “TIO AND persistence”, “TIO AND persistent”, “TIO AND untreated”, “TIO AND unoperable”, “TIO AND unresectable”, TIO AND undetected”, along with published TIO case series.

General data, clinical profile, location of tumor, histopathology, and treatment and management of persistence/recurrence of disease, were collected for each patient, if available.

## Results

### Cross sectional and retrospective study

Patients with recurrent, persistent TIO and affected patients with not operable or not detected tumor treated in seven major Italian referral centers for metabolic bone diseases were recorded, as re-evaluated during the period from February through September 2020, to assess last outcome. Sixteen Caucasian patients with these characteristics were collected, with a female: male ratio = 1:1 (Table 1a, b). All women were in their postmenopausal state (mean age at menopause: 50 ± 2.6 years; mean years since menopause at first evaluation: 16 ± 11.9).

**Table 1:** Data referred to the Italian cohort of patients with persistent (P), recurrent TIO (R), or TIO with not localized/not operable tumor (NLo) are shown (NA not assessed);**1a:** demographics and clinical characteristics (a = Gaetano Pini Institute Milano, Rheumatology Unit; b = University Hospital of Florence, Bone Metabolic Diseases Unit; c = University Hospital of Padova, Department of Medicine; d = University Hospital of Verona, Internal Medicine; e = University Hospital of Verona. Rheumatology Unit; f = University Hospital of Pisa, Rheumatology Unit; g = University Hospital of Siena, Internal Medicine; F = female. M = male) Adequate calcium intake: 1000 mg/day for men, 1200 mg/day for women (according to Institute of Medicine recommendations for the considered age range). **1b:** Biochemical and radiological data. Tumor site and histology. Treatments and outcome (i = intact FGF23 assay pg/ml, c = C-terminal FGF23 assay pmol/L)

Patient	Referral center	Classification	Sex	Age at onset (years)	Symptoms at onset	Age at diagnosis (years)	Age at (re)evaluation (years)	Muscle pain at evaluation	Bone pain at evaluation	Fractures (sites)	Pseudo-fractures	Weight-loss (Kg)	Height-reduction (cm)	Bone deformity, kyphosis	Waddling gait	Adequate calcium intake*
1a																
1	a	P	F	44	Diffuse muscle and bone pain, fatigue	46	53	Yes	Yes	Ribs, bilateral hip	No	Yes [10]	No	No	Yes	Yes
2	a	P	F	55	Diffuse muscle and bone pain, fatigue	58	75	Yes	Yes	Ribs, bilateral humerus	Pelvis	Yes [4]	No	Yes	No	No
3	a	P	M	32	Diffuse muscle and bone pain, fatigue	37	45	Yes	Yes	Ribs, Spine (multiple)distal radius	No	Yes [12]	Yes [5]	No	Yes	Yes
4	a	R	F	50	Diffuse muscle and bone pain, fatigue	54	71	Yes	Yes	Ribs, hip	No	Yes [11]	No	No	Yes	Yes
5	a	R	F	33	Diffuse muscle and bone pain, fatigue	35	55	Yes	Yes	Bilateral distal femur, spine (single), tibia, heel	Pelvis	Yes [7]	Yes [3]	No	Yes	Yes
6	a	R	F	64	Diffuse muscle and bone pain, fatigue	65	80	Yes	Yes	Ribs, sacrum, tibia	No	Yes [10]	No	No	Yes	Yes
7	b	NL	M	42	Diffuse muscle, joint, and bone pain, fatigue	45	54	No	Yes	Foot finger	No	No	No	No	yes	Yes
8	b	NL	F	50	Diffuse muscle, joint, and bone pain, fatigue	51	56	Yes	Yes	No	Pelvis	No	No	No	No	Yes
9	b	R	M	36	Diffuse muscle and bone pain, fatigue, nosebleed	40	54	Yes	Yes	Ankle	Pelvis	No	No	No	Yes	
10	c	P	M	46	Diffuse muscle and bone pain, fatigue	48	53	No	Yes	Ribs, bilateral hip	No	No	No	No	No	Yes
11	c	R	M	58	Diffuse muscle, joint and bone pain, fatigue	61	66	Yes	Yes	Ribs	No	No	No	No	No	Yes
12	d	P	F	53	Diffuse muscle and bone pain, fatigue, fractures	54	56	Yes	Yes	Spine (multiple)	No	No	Yes [5]	Yes	No	Yes
13	d	NL	M	57	Diffuse muscle and bone pain, fatigue	58	59	No	Yes	Spine (multiple)bilateral ankle	No	No	Yes [5]	Yes	No	Yes
14	e	R	M	65	Diffuse muscle, joint, and bone pain. fatigue; algodystrophy	66	88	No	Yes	Spine (single)	No	No	No	No	No	Yes
15	f	R	M	51	Diffuse muscle, and bone pain, fatigue; fracture	54	60	yes	Yes	Spine (single)	No	No	Yes [5]	No	No	No
16	g	P	F	64	Diffuse muscle and bone pain, fatigue; waddling gait	68	82	Yes	Yes	Wrist, spine (multiple), femur	No	No	No	Yes	No	Yes

Patient	L1-L4 T-score Z-score	Femoral Neck T-score Z-score	Serum Phosphate (mg/dl)	Serum Total ALP (IU/L)	Serum Bone ALP (mcg/L)	Serum FGF-23 (pg/ml or pmol/L)	Serum 25(OH)D (ng/ml)	Daily treatment at diagnosis	TIO-related tumor localization	Localization technique	Age at tumor localization	First Treatment	Histology	Second treatment	Third treatment
1b															
1	NA	NA	1.2	NA	NA	117 (i)	41	Calcitriol (0,5 mcg)	Presacral area	X rays, ^68^Ga-DOTA PET, MRI, CT	46	Presacral affected tissue removal; incomplete resection	PMT	Explorative laparotomy without tumor localization	Burosumab
2	−3.1 −1.7	−2.2 −1.6	1.6	NA	NA	NA	32	Calcitriol (0,5 mcg)	Proximal humerus	X rays, Octreoscan, MRI	59	Curettage of bone lesion; incomplete resection	PMT	Phosphate salts and calcitriol	
3	−2.8 −1.5	−2.3 −1.0	0.8	NA	NA	270 (i)	65	Phosphate salts (2000 mg) + calcitriol (0,25 mcg)	Frontal sinus	Octreoscan, ^68^Ga-DOTA PET, MRI, CT	39	Craniotomy; incomplete resection	PMT	Phosphate salts and calcitriol	
4	−0.7 0.2	NA	1.1	NA	NA	2261 (i)	11	Phosphate salts (1000 mg) + calcitriol (0,25 mcg)	Distal femur	Octreoscan	58	Curettage of the femur and reconstruction; complete resection	PMT	Reoperation, thermoablation, and reconstruction	Phosphate salts and calcitriol
5	−2.7 −1.3	NA	0.7	NA	NA	NA	24	Phosphate salts (1000 mg)	Maxillary sinus	Octreoscan, MRI, CT	36	Nasal sinus mucosa removal and adjacent bone curettage; complete resection	PMT	Reoperation and reconstruction	Phosphate salts and calcitriol
6	−3.6 −1.8	NA	1.5	NA	NA	NA	28	Calcitriol (0,25 mcg)	Rib and adjacent lung	X Rays, CT	64	Rib resection and upper lung lobectomy; complete resection	PMT	Phosphate salts and calcitriol	
7	NA	NA	1.5	350	90.8	NA	NA	Phosphate salts (1500 mg)	NL	Bone scintigraphy, PET/CT scan, Octroscan	43	Phosphate salts and calcitriol (surgery not performed)	--	Burosumab	
8	−2.2 −1.4	−2.07 −1.0	1.5	220	67.9	65 (i)	NA	Phosphate salts (1500 mg) + calcitriol (0,50 mcg)	NL	Octreoscan, ^18^F-FDG PET, ^68^Ga-DOTA PET	51	Phosphate salts and calcitriol (surgery not performed)	--	Burosumab	
9	−6.1 −3.9	−4.1 −2.9	0.8	1252	95.9	>600 (i)	NA	No	Frontal and ethmoidal sinuses, adjacent meninges	MRI, Octreoscan	40	Craniotomy; partial removal of frontal bone; incomplete resection	PMT	Transphenoidal resection	Phosphate salts and calcitriol
10	−2.2 −1.9	−3.6 −1.9	0.6	540	NA	2.93 (c)	50	Phosphate salts (1500 mg) + calcitriol (1 mcg)	Proximal tibia	^18^F-FDG PET, ^68^Ga-DOTA PET	49	Proximal tibial bone lesion removal	(Not conclusive)	Reoperation	Phosphate salts and calcitriol
11	NA	NA	1	NA	80.7	5.07 (c)	30	Phosphate salts (2500 mg) + calcitriol = ,25 mcg)	Patella	^68^Ga-DOTA PET	61	Retropatellar lesion removal; complete resection	PMT	Phosphate salts and calcitriol	
12	−3.6 −2.2	−2.6 −1.5	2.28	NA	NA	5.36 (c)	23	Phosphate salts (2000 mg) + calcitriol 0,50 mcg)	Brain., paramedian	^68^Ga-DOTA PET, angio-MRI	NA	Gamma-knife radiosurgery	--	Phosphate salts and calcitriol	
13	0.70 1.1	−1.0 0.9	1.17	NA	NA	3.60 (c)	27	Phosphate salts (3000 mg) + calcitriol 1 mcg)	Brain, parietal	^68^Ga-DOTA PET, angio-CT	58	Not performed	--	Phosphate salts and calcitriol	
14	−4.01 −2.4	−3.2 −2.0	1.4	168	NA	NA	14	Phosphate salts (2000 mg)	Forearm	Ultrasound, CT	69	Bone lesion removal; complete resection	Tissue of glandular origin	Reoperation	Phosphate salts and calcitriol
15	−3.4 −2.1	−3.0 −1.9	1.1	NA	64	NA	12	Phosphate salts (3000 mg) + calcitriol (1 mcg)	Frontoethmoidal bone	Bone scintigraphy, CT, ^68^Ga-DOTA PET	55	Frontoethmoidal bone lesion removal with reconstruction of basal skull; complete resection	Giant cell tumor		
16	−2.0 0.4	−3.6 −1.8	0.9	NA	117	1824 (i)	12	Phosphate salts (1500 mg) + calcitriol (1 mcg)	Sacrum	Bone scintigraphy, CT, Octreoscan	69	Sacral bone lesion removal; incomplete resection	Mesenchymal tumor with chondromyxoid features		

The mean prevalence of patients with recurrent/persistent TIO or diagnosed TIO without tumor localization, considering all patients with TIO, thus including cured TIO patients, was 54.2 ± 36.2%, ranging between 25% and 100% (data referred to patients with cured TIO are not shown).

Mean age for the onset of symptoms was 50 ± 10.6 years, while mean age at diagnosis was 52.5 ± 10.1 years, with a consequent delay in diagnosis of 2.5 ± 1.3 years (range: 1–5 years). Initial symptoms developed in a relatively short period of time (few months) and consisted mostly of aspecific generalized bone and muscle pain and fatigue for all patients. Joint discomfort or pain at onset were reported by 4 patients (25%), one of whom experienced a complex regional pain syndrome of the foot. Fragility fractures were reported in 2 patients (12.5%) as one of the first symptoms of the disease.

**Fig. 1:**
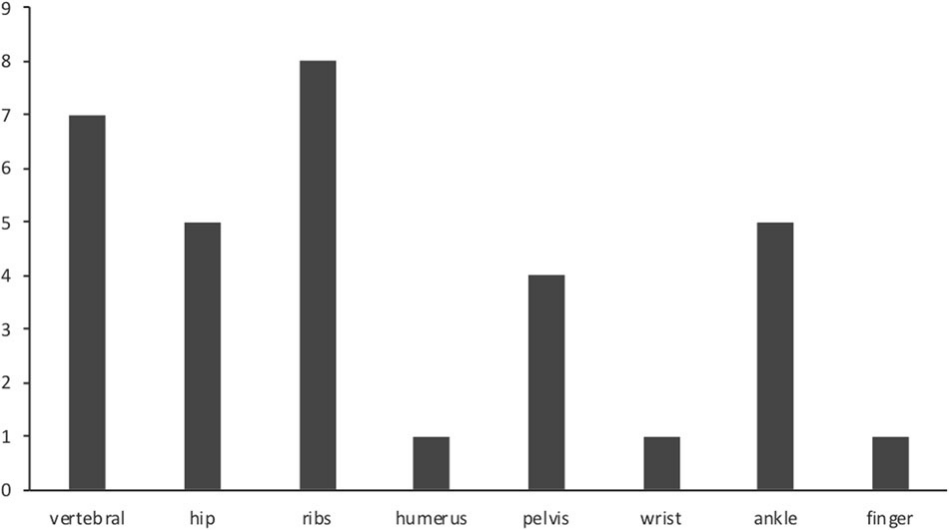
Site of pathologic fractures. Site of reported fractures in the Italian cohort of patients with persistent (P), recurrent TIO (R), or TIO with not localized/not operable tumor (NLo) (cumulative count)

The mean age at evaluation was 62.9 ± 12.6 years. At the time of the cross-sectional study, TIO-related symptoms (muscle pain, bone pain, fragility fractures, pseudofractures) were systematically assessed. Disabling, often generalized, bone pain was reported by all patients, while myalgia or proximal muscle weakness was present in 12 patients (75%). All patients were positive for prevalent or reported fragility fractures or pseudofractures, which usually occurred 1–2 years after the onset of generalized symptoms. As shown in Fig. 1, most were rib and vertebral fractures, often multiple, followed by hip fractures (bilateral hip fractures in 3 cases, atypical femoral fracture in one patient) and ankle fractures. Pelvic pseudo-fractures were also reported, while fractures of upper limbs were rare. Besides specific, TIO-related symptoms, six out of 16 patients (37.5%) experienced significant weight loss from baseline (mean 9 ± 3 Kg) within 1–4 years; in 5 patients, significant height reduction was demonstrated because of multiple vertebral fractures. At physical examination, kyphosis and rib cage deformity was present in 4 patients, while typical waddling gait was present in 7 patients (43.7%).

Calcium intake (without or with supplementation) was adequate (i.e., 1000 mg/day for men, 1200 mg/day for women, according to Institute of Medicine recommendations for the considered age-range) in 13 out of 16 patients (81,2%). Familial history was negative for diseases of bone and mineral metabolism in all patients. No relevant comorbidities were associated. With respect to other proliferative diseases, one patient had essential thrombocythemia, one a melanoma, one a meningioma, one a gastric adenocarcinoma, and one a cervical adenocarcinoma, all appropriately cured.

Bone mineral density (BMD), as expressed in terms of T-score, was markedly reduced both at lumbar spine and femoral neck (mean T-score at lumbar spine −2.7 ± 1.7, mean T-score at femoral neck −2.7 ± 0.9).

Phosphatemia was markedly reduced (1.2 ± 0.4 mg/dl) due to phosphate wasting (reduced TmP/GFR), total alkaline phosphatase and/or bone alkaline phosphatase, when assessed, were significantly increased (Table 1b), while the other parameters related to mineral metabolism, such as calcemia, calciuria, serum PTH and 1,25(OH)_2_D, were normal for most patients (not shown). Serum intact or C-terminal FGF23 was measured in 10 patients and was found to be increased (Table 1b).

Serum 25(OH)D was normal (above 20 ng/ml) in 12 patients (75%) who received proper cholecalciferol supplement, while 4 patients (25%) were vitamin D deficient (serum 25(OH)D less than 20 ng/ml).

At the time of diagnosis, almost all patients (14 out of 16 patients, 68.7%) were placed under therapy with phosphate salts (1200–3000 mg/day) and/or calcitriol (0-25-2 mcg/day); supplementation with cholecalciferol and/or calcium carbonate was advised in deficient patients.

**Fig. 2:**
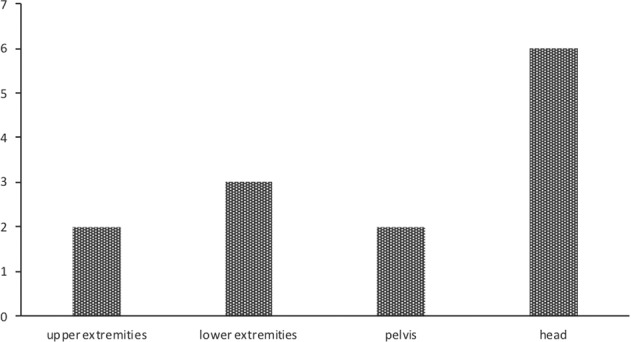
Mesenchymal tumor site. Site of identified TIO-related tumor in the Italian cohort of patients with persistent (P), recurrent TIO (R), or TIO with not localized/not operable tumor (NLo)

In 3 patients (18.8%), the FGF-23 secreting tumor was not operable or not localized by commonly employed nor advanced techniques of nuclear medicine imaging (indicated as NL); in 7 patients (43.7%), TIO recurred 6 months after first surgical treatment (indicated as R); and in 6 patients (37.5%) the disease was persistent after specific treatment (i.e., persistent hypophosphatemia or hypophosphatemia recurrence within 6 months after treatment, as specified above, indicated as P). In cases in which the tumor was localized at baseline, the most common site was the head (in particular, paranasal sinuses) (Fig. 2). Successful localization techniques were primarily ^68^Ga-DOTA (5 out of 13 cases, 38.4%) or OctreoScan associated with MRI or angio-MRI (4 cases, 30.8%). In 4 cases, the tumor was localized within one year after diagnosis; in 9 cases the tumor was localized 1–4 years after diagnosis (mean 2 years).

Surgery was performed in all R and P cases. In one of the P cases, gamma knife radiosurgery was carried out. At histological examination, resected tumor mainly consisted of PMT with hemangiopericytoma features.

In P cases (*n* = 6), TIO symptoms and/or hypophosphatemia persisted after surgery or radiosurgery. In 5 of these cases, surgical treatment was already considered incomplete at the time of surgery. Hypophosphatemia persisted unchanged in the follow-up, while FGF23 was confirmed increased; TIO-related symptoms did not resolve. Patients were then maintained under therapy with phosphate salts and calcitriol. For two patients, additional surgery was considered feasible and was then performed, although not radical and unsuccessful. One of these two patients was then placed under burosumab therapy, with achievement of normophosphatemia and control of symptoms.

In R cases (*n* = 7), TIO symptoms and/or hypophosphatemia recurred 6 months after surgery (mean 52,7 ± 28.7 months, range 12–82 months). Four patients underwent further surgery after restadiation for local tumor recurrence, which was curative in 3 (75%). In 3 of 4 patients with recurrent disease in which second surgery was not curative or not performed, therapy with phosphate salts and calcitriol was restarted. None of these patients were placed under burosumab therapy.

In two of the NL patients (*n* = 3), treatment with anti-FGF23 antibody was started (compassionate use), achieving full control of symptoms in a period of 2–6 months, with normalization of phosphatemia in one subject (data not shown).

### Literature review

Data were retrieved from 55 different publications [25–73]; data duplication was avoided. This cohort includes 216 patients with recurrent/persistent disease or with not localized/not operable tumors.

Relevant data are provided in Supplementary Tables 1–3, for patients with persistent disease (Pe), recurrent disease (Re), and individuals with not localized/not operable tumors (NLo), respectively.

Clinical details are summarized in Table 2. Due to heterogeneity in reporting various parameters, the total number of cases included has been specified for each analyzed index.

**Table 2:** Data referred to patients with persistent (Pe), recurrent (Re) and not localized/not operable (NLo) TIO as retrieved from a systematic review of the literature: demographics, clinical characteristics, tumor site and histology, treatments and outcome of patients are shown (*Patient’s decision, RT Radiotherapy; CHT Chemotherapy; NA Not available)

		PERSISTENCE (Pe) (*N* = 77)		RECURRENCE (Re) (*N* = 59)		NOT LOCALIZED/NOT OPERABLE (NLo) (*N* = 80)	
		Mean ± SD	No of patients with available data	Mean ± SD	No of patients with available data	Mean ± SD	No of patients with available data
MEDIUM AGE AT DIAGNOSIS		44.94	54	49.46	28	41.15	26
GENDER	Male	30 (55.55%)	54	10 (32.25%)	31	15 (57.69%)	26
	Female	24 (44.45%)		21 (67.75%)		11 (42.31%)	
DURATION OF SYMPTOMS (years)		7.51	38	10.95	35	NA	NA
HISTOPATHOLOGY (PMT/PMTMCT)		37 (68.51%)	54	33 (82.50%)	40	-	-
LOCALIZATION OF TUMOR	Low extremities	20 (37.03%)	54	25 (60.97%)	41	-	80
	Vertebral column	2 (3.70%)		4 (9.75%)		-	
	Head and neck region	25 (46.29%)		10 (24.39%)		-	
	Other	7 (12.96%)		2 (4.87%)		-	
	Not localized	-		-		74 (92.5%)	
	Not operable	-		-		4 (5%) + 2 (2.5%)*	
TREATMENT	Surgery	27 (54%)	50	33 (80.48%)	41	-	15
	Curettage/incomplete resection	18 (36%)		5 (12.19%)		-	
	Surgery + RT	1 (2%)		2 (4.87%)		-	
	Other	4 (8%)		1 (2.43%)		1 burosumab (6.66%), 13 only oral supplementation (86.66%), 1 other (6.66%)	
TIME FREE FROM RECURRENCE (months)		-	-	41.92	20	-	-
SECOND TREATMENT	Surgery	14 (31.81%)	44	10 (40%)	25	-	-
	Curettage/incomplete resection	5 (11.36%)		0 (0%)		-	
	Surgery + RT	4 (9.09%)		2 (8%)		-	
	RT	1 (2.27%)		1 (4%)		-	
	CHT	1 (2.27%)		1 (4%)		-	
	Oral phosphate only	11 (25%)		6 (24%)		-	
	Other	8 (18.18%)		5 (20%)		-	
CURED		12 (52.17%)	23	9 (33.33%)	27	-	-

From a histopathological point of view, the phosphaturic mesenchymal tumors mixed connective tissue variant (PMT/PMTMCT) was the most frequently reported tumor type, being 68.5% and 82.5% of cases in patients with Pe and Re, respectively. The remaining rare types of tumors are listed in detail in Supplementary Tables 1–3.

Regarding patients with Pe (*n* = 77), the mean age at diagnosis was 44.7 ± 15.2 years, with a prevalence of male subjects (52.9%). Delay in diagnosis experienced was 7.5 ± 5.5 years. The frequency of tumor sites, in descending order, were head and neck region, lower extremities, vertebral column, and others. Although for most of the patients (54%) a complete resection of the tumor was documented, TIO-related symptoms persisted or recurred within 6 months after surgical treatment. These patients were then managed with subsequent surgery (31.81%) or other treatments (radiotherapy or chemotherapy). Eleven patients (25%) underwent only oral phosphate supplementation. Of the 23 patients whose outcome is available, only 12 (15.7% of total Pe cases) with persistent disease were cured after the second treatment.

Regarding patients with Re (*N* = 59), the mean age at diagnosis was 48.8 ± 12.9 years, with a prevalence of female subjects (66.6%). Delay in diagnosis experienced was 11.0 ± 9.0 years. The frequency of tumor sites, in descending order, were low extremities, head and neck region, vertebral column, and others. Even if a complete resection of the tumor was performed for most of the patients (80.5%) at the time of first surgery, symptoms of TIO relapsed after a mean time free from disease of 42.7 ± 33.9 months. Most of these patients were managed with a second surgical treatment (40%). A minority of patients were managed with other treatments, such as radiotherapy or chemotherapy. Just one fourth of patients (6 patients, namely 24%) began oral phosphate supplementation. Nevertheless, only 9 patients (15.2% of total Re cases) with recurrent disease received a treatment after the second surgery. In most of these patients, second treatment was not radical, and the disease therefore became persistent.

In NLo patients (*n* = 80), the mean age at diagnosis was 41.1 ± 17.1 years, with a prevalence of male subjects (57.7%). Delay in diagnosis was not specified for most of the patients in this group. In most of these individuals (92.5%), the tumor was not localized, even if advanced nuclear imaging was performed. In 4 patients (5%), the tumors were localized but not operable, while 2 patients (2.5%) chose not to have surgery, even if the tumor was localized and considered operable. Most of these patients receive oral phosphate supplementation as a treatment (86.7%). Only one patient received therapy with burosumab.

## Discussion

This study, for the first time, focuses on persistent, recurrent TIO, and patients with not operable/not localized TIO-related tumors. It also performs a systematic review of the literature, identifying cases with similar characteristics within the case series, or case reports published so far. Previously published papers have often described TIO cases without details of the disease, which are fundamental to better understand its pathophysiology, an evolving clinical picture, in order to plan proper treatment or re-treatment.

As demonstrated in the national case series herein described, for patients in which a tentative surgical approach is undertaken, the disease persists or recurs in a significant percentage of TIO patients (54.2 ± 36.2 %). This percentage is rather high with respect to data shown in previous literature. This can be explained by the fact that the study herein presented is not a prospective trial and affected population is heterogenous for assessment procedures among different Centers.

Although hypophosphatemia-related disorders were identified relatively recently [6], they already constitute an important chapter in mineral and skeletal metabolism and pathophysiology [4]. With the identification of FGF23 as the main regulator of phosphate homeostasis and the recent introduction of automated FGF23 assays, diagnosis should be relatively easier than in the past. Nonetheless, while patients with congenital forms of hypophosphatemia are usually correctly identified in specialist pediatric settings due to typical skeletal abnormalities (i.e., hypophosphatemic rickets), adult forms of hypophosphatemia, which are characterized by rapid marked deterioration of quality of life, along with osteomalacia-related signs and symptoms (generalized bone and muscle pain), are still a challenge for the physician, due to their aspecific symptoms [13]. In the absence of systematic evaluation, real prevalence and incidence of TIO in the general population remain to be ascertained [3]. Since symptoms are aspecific, in the absence of serum phosphate assessment, which usually occurs in specialistic centers (mainly rheumatology and endocrinology units), these patients often experience a delay in diagnosis, as shown by literature review [1, 14, 25–75], confirmed by the findings of the cross-sectional and retrospective study described in this paper. Although fractures may be one of the first signs of the disease in some patients, with the clinical picture at onset mainly characterized by diffuse bone pain, fatigue, and muscle weakness, skeletal fragility is a constant feature when systematically assessed, as demonstrated in our case series, with multiple fractures occurring 1–2 years after the onset of generalized symptoms. Indeed, bone mineral density, as expressed in terms of T-score, was markedly reduced both at lumbar spine and femoral neck, reflecting generalized osteomalacia. While the most common fractures affected vertebral bodies and ribs, it is worth noting that there were also bilateral femoral fractures, reinforcing the importance of the assessment of serum phosphate as first-level examination after a hip fracture or other major fragility fractures, even in older subjects. In addition to height loss (mainly due to vertebral fractures), weight loss, present in up to one third of the described patients, is also an important sign to consider, along with waddling gait and thorax deformity.

Once the disease has been diagnosed, patients usually experience a further delay in treatment, as demonstrated by the case series. In the individuals described, the delay of diagnosis (2.5 ± 1.3 years), although lower than that found in the review of the literature (7.5–11 years), is often associated with a delay in treatment, since TIO-related tumors are often difficult to localize. Even when advanced imaging techniques, such as OctreoScan or ^68^Ga, are applied, the tumor sometimes is not located.

Although replacement therapy with phosphate salts and calcitriol does not cure the disease, which in fact remains mainly symptomatic, this is usually the first line of treatment. Surgery, the therapy of choice, relies not only on effective tumor localization, but also on radical resection. As demonstrated in our case series, recurrences and/or persistency are common. Although morphological atypies not are usually found at histological examination, PMT tumors typically recur [15]. When further local treatment is planned because of an incomplete resection or recurrence, it very rarely leads to resolution of the disease.

An anti-FGF23 monoclonal antibody, burosumab, has recently been shown to be safe and effective in controlling signs and symptoms in pediatric and adult patients with hypophosphatemic rickets due to FGF-23 excess. It has also been tested in adults with TIO, with promising results [22–24].

In our case series, only 3 patients have been placed under burosumab therapy obtained for compassionate use. In these patients, burosumab was able to correct symptoms and improve quality of life. Burosumab should be proposed for long-term therapy in R and P cases, avoiding recurrent surgeries, which are often destructive and not curative, as well as for patients in which the tumor has not been localized or is not operable.

While we acknowledge that this study has limitations because it does not include all the Italian patients with persistent or recurrent TIO, it is the first survey of its kind of this rare disorder. Compared to similar TIO cases retrieved from the literature, this 16-case series adds an important contribution to the field, considering the systematic assessment of all the features described, many of which were not taken into consideration in previous publications.

## Conclusion

TIO is a rare disease of mineral metabolism caused by phosphate imbalance due to FGF-23 excess. Recurrent and persistent cases constitute an important portion of affected patients and pose key treatment issues, since replacement therapy with phosphate and calcitriol does not fully correct this systemic disorder. While surgery is still the therapy of choice for this disease once the tumor has been localized, and must be pursued as a first line of treatment, difficulties in tumor localization and high probability of local recurrence undermine this procedure. Burosumab, an anti-FGF23 monoclonal antibody, could be proposed to patients with recurrent or persistent TIO when surgery fails or is not feasible. Further open-label studies in these TIO patients are necessary to confirm the efficacy of this treatment and its safety, also in the long-term.

## Supplementary Information

Supplementary tables
