# Supplementary material for: Persistence and recurrence in tumor-induced osteomalacia: A systematic review of the literature and results from a national survey/case series
Source: Endocrine. 2022 Apr 5;76(3):709–21. doi: 10.1007/s12020-022-03039-2 (PMC9156492; doi:10.1007/s12020-022-03039-2)
Supplement: Supplementary file 1 — Supplementary tables [file 12020_2022_3039_MOESM1_ESM.docx]

**Supplementary table 1:** demographics, clinical characteristics, tumor site and histology, treatments and outcome of patients with persistent TIO (Pe), as retrieved from the literature, are shown (M=male; F=female; na=not available data; CHT=chemiotherapy; RT=radiotherapy; FU=follow up).

|  | **N° of cases** | **AGE at diagnosis** | **SEX** | **CLINICAL PRESENTATION** | **DELAY IN DIAGNOSIS (years)** | **HYSTOPATHOLOGY** | **TUMOR LOCALIZATION** | **FIRST TREATMENT** | **TREATMENT of PERSISTENT DISEASE** | **STATUS** | **COMMENTS** |
| --- | --- | --- | --- | --- | --- | --- | --- | --- | --- | --- | --- |
| Ref #25 | 4 | 46 | M | bone pain, muscle weakness, fractures | 13 | Hemangiopericytoma | left petrous tumor | surgery | Radiotherapy | cured | **-** |
|  |  | 60 | M | bone pain, muscle weakness, fractures | 1 | PMTMCT | left ethmoid sinus | surgery (endoscopic surgery) | surgery (endoscopic surgery) + intensity-modulated radiation therapy | cured | **-** |
|  |  | 39 | M | bone pain, muscle weakness | 4 | PMTMCT | right frontal and ethmoid sinus | surgery | surgery (endoscopic surgery) | cured | **-** |
|  |  | 53 | F | pain, muscle weakness | 3 | PMTMCT | base of the skull | surgery | surgery + Peptide receptor radionuclide therapy | persistence | **-** |
| Ref #26 | 6 | 28 | M | na | na | HPT | head of left fibula | surgery | oral supplementation | na | **-** |
|  |  | 26 | F | na | na | GCT | right nasal cavity | surgery | oral supplementation | na | **-** |
|  |  | 30 | M | na | na | GCT | left thigh | surgery | oral supplementation | na | **-** |
|  |  | 67 | M | na | na | PMT | right medial femoral condyle and S2 vertebral body | surgery | oral supplementation | na | **-** |
|  |  | 56 | F | na | na | PMTMCT | right pelvic blade | surgery | oral supplementation | na | recurrence within 6 months from first surgery |
|  |  | 36 | F | na | na | HPT (lipomatous type) | left nasal cavity | surgery | oral supplementation | na | recurrence within 6 months from first surgery |
| Ref #27 | 1 | 40 | M | muscle pain, pseudofractures | 10 | PMT | left popliteal fossa, left proximal tibiofibula soft tissue, right adductor magnus | surgery | multiple surgery | na | **-** |
| Ref #28 | 3 | 53 | M | na | 7 | PMT | lateral epicondyle of the femur | curettage + HSB (high-speed burring) | curettage | na | **-** |
|  |  | 62 | M | na | 5 | PMT | L1 | curettage and segmental resection and posterior fusion | curettage and anterior fusion | na | **-** |
|  |  | 69 | F | na | 25 | PMT | ischium | curettage | na | na | **-** |
| Ref #29 | 6 | 19 | M | bone pain, fatigue, fractures | 1.5 | PMT | ulnar shaft | surgery | na | na | **-** |
|  |  | 50 | M | bone pain, fatigue, fractures | 6 | PMT | Tibial plateau | curettage | surgery (no complete resection) | na | **-** |
|  |  | 27 | M | bone pain, fatigue, fractures | 7 | PMT | Greater trochanter of femur | curettage | curettage | na | **-** |
|  |  | 22 | F | bone pain, fractures | 3 | 1° negative, 2° PMT | femoral head | curettage | surgery (complete resection) | na | **-** |
|  |  | 59 | F | bone pain, fractures | 14 | PMT | Medial femoral epicondyle | curettage | surgery | na | recurrence after 5 months |
|  |  | 32 | M | bone pain, weakness, fractures | 5 | PMT | Greater trochanter of femur | curettage | na | na | recurrence after 5 months |
| Ref #30 | 2 | 65 | F | fatigue, bone pain, muscle weakness, fractures | 12 | PMT | lower leg | surgery | multiple surgery, octreotide | na | Lung metastasis |
|  |  | 74 | M | back pain, fatigue, bone pain, muscle weakness, stress fractures | 6 | PMT | calcaneus | curettage and bone graft | observation (asymptomatic) | na | **-** |
| Ref #14 | 3 | 29 | F | pain, weakness, fractures | na | PMT | Right maxilla | surgery with margins of the resected tissue positive for tumor | oral supplementation | na | **-** |
|  |  | 51 | M | pain, fractures, weakness | na | Features consistent with PMT and ameloblastic fibrosarcoma | Right mandible | previously treated in another hospital | oral supplementation | na | Lung metastasis |
|  |  | 31 | F | rickets in childhood, weakness, amputation | na | PMT | left forearm with pulmonary metastases | previously treated in another hospital | CHT | died | **-** |
| Ref #31 | 4 | 29 | F | TIO | 6 | PMT | Foot | Surgery* | na | na | *Incomplete resection of tumor |
|  |  | 21 | M | TIO | 7 | PMT | Elbow | Surgery* | na | na | *Incomplete resection of tumor |
|  |  | 67 | M | TIO | 9 | PMT | Scapular region | Surgery* | na | na | *Incomplete resection of tumor |
|  |  | 59 | F | TIO | 14 | PMT | Leg | Surgery* | na | na | *Incomplete resection of tumor |
| Ref #32 | 1 | 55 | M | muscle weakness, bone pain | na | benign mesenchimal tumor | sacrum | surgery | surgery | cured | **-** |
| Ref #33 | 1 | 22 | M | diffuse bone pain | 8 | HPC | right sphenoid wing | embolization + surgery | embolization + multiple surgery (no definitive resection of the lesion) + octreotide + dasatinib* | na | * high expression of PDGFR |
| Ref #34 | 1 | 59 | F | fractures, pain, unable to walk | 10 | haemangiopericytoma | vulva | Surgery* | oral supplementation | na | *Incomplete resection of tumor |
| Ref #35 | 1 | 32 | M | Intracranial hemorrhage (x2); hip and low-back pain | 1 | PMTMCT | Left anterior fossa, ehtmoid sinus and nasal cavity | multiple surgery | multiple surgery | na | **-** |
| Ref #36 | 1 | 29 | M | Lumbar, knee and foot pain, muscle weakness (confinement in bed) | 2 | osteosarcoma | Mandible | surgery | surgery, RT, CHT | cured | **-** |
| Ref #37 | 1 | 43 | F | bone pain, muscle weakness, difficulty in walking, esotropia in right eye and horizontal diplopia | 4 | PMTMCT | apex partis petrosae ossis temporalis with sinus cavernous infiltration and internal carotid artery surrounded partially in the right side | Surgery* | octreotide | na | *****The tumor was only resected grossly |
| Ref #38 | 1 | 22 | M | fractures, wheel-chair bound | 2 | haemangiopericytoma | vault of rhinopharynx, involving ethmoid and sphenoid sinuses | surgery | surgery | cured | **-** |
| Ref #39 | 1 | 56 | M | rib pain, fractures, osteomalacia | 19 | ameloblastic fibrosarcoma | right mandibular body | surgery* | multiple surgery + oral supplementation** | pulmonary metastases in FU; patient is asymptomatic with oral supplementation | *tumor was focally present at the surgical margin, however serum phosphorus level returned to normal without oral supplementation for 4 years  ** persistent disease due to pulmonary metastasis, stable at imaging |
| Ref #40 | 1 | 49 | F | bone pain, muscle weakness | 18 | PMT | right mandible | Surgery* | multiple surgery (seven times) | alive with disease (20 years of FU) | *Incomplete resection of tumor |
| Ref #41 | 1 | 52 | M | muscle weakness, inability to rise from a seated position, fractures | several years | PMT | left foot | Surgery* | na | na | *Incomplete resection of tumor |
| Ref #42 | 1 | 40 | M | severe weakness | 2.5 | PMT | right tarsal region | surgery | surgery, octreotide | metastasis | **-** |
| Ref #43 | 24 | na | na | na | na | na | na | na | na | na | **-** |
| Ref #44 | 1 | 69 | M | bone pain, myalgia, muscle weakness, fractures | na | PMT | right humerus | surgery | Surgery* | na | *2 local recurrences with hypophosphatemia within 3 months after surgery |
| Ref #45 | 1 | 53 | F | bone pain, walk instability | 2 | PMT | base of the skull and left basiocciput | surgery | 1° surgery; 2° PRRT with 177Lu-DOTATATE; oral supplementation | cured | 1° recurrence after 4 months from surgery; 2° recurrence: 7 months |
| Ref #46 | 1 | 60 | F | femur neck fracture | 6 | 1° spindle cell hemangioma; 2° glomangiopericytoma | right maxillary sinus | Surgery* | surgery (partial resection due to position near maxillary artery) | cured | *Incomplete resection of tumor |
| Ref #47 | 1 | 39 | F | weakness, fatigue, waddling gait, and lower back pain | 3.5 | PMT with prominent giant cell and vascular components | right maxillar sinus | Surgery* | surgery | cured | *recurrence after 10 weeks |
| Ref #48 | 1 | 63 | F | diffuse bone pain, muscle weakness, difficulty walking, pseudofractures | 5 | haemangioperycitoma | left maxillary sinus | Surgery* | surgery + radiotherapy | persistence of symptoms and hypophosphatemia; died for bronchopneumonia | * Incomplete resection of tumor; recurrence after 3 months from surgery |
| Ref #49 | 1 | 42 | F | back pain | 9 | PMTMCT | right mandible | Surgery* | oral supplementation | asymptomatic | *Incomplete resection of tumor |
| Ref #50 | 1 | 59 | F | bone pain, fatigue, fractures | 2 | meningioma (with mesenchymal chondrosarcoma cells) | olfactory groove on the right side | surgery (microsurgical resection)* | surgery (microsurgical resection) | na | *recurrence after 2 months from surgery |
| Ref #51 | 1 | 36 | F | bone and muscle pain | 9 | PMTMCT | ethmoid sinus | Surgery* | surgery | cured | *Incomplete resection of tumor |
| Ref #52 | 1 | 40 | F | pain, difficulty in walking | 1 | hemangiopericytoma | left nasal cavity | surgery +oral supplementation | surgery | cured | **-** |
| Ref #1 | 2 | 27 | F | TIO | na | benign mesenchymal tumor | tibia | na | na | na | **-** |
|  |  | 30 | M | TIO | na | PMTMCT | ischium | na | na | na | **-** |
| Ref #53 | 1 | 42 | M | TIO | 8 | PMT | right nasal cavity and ethmoid sinus, intracranial invasion | surgery (previously incomplete endoscopic resection + post-operative local radiotherapy at another institution, without clinical remission) | surgery | recurrence after 1 year, died of a brain hernia 2 years later | **-** |
| Ref #54 | 1 | 49 | M | weakness, bone pain | na | PMT | L5 | surgery + oral supplementation | surgery | Cured | - |

**Supplementary table 2:** demographics, clinical characteristics, tumor site and histology, treatments and outcome of patients with persistent TIO (Re), as retrieved from the literature, are shown (M=male; F=female; na=not available data; CT = computed tomography; MRI = magnetic resonance imaging).

|  | **N° of cases** | **AGE at DIAGNOSIS** | **SEX** | **DELAY IN DIAGNOSIS (years)** | **CLINICAL PRESENTATION** | **HYSTOPATHOLOGY** | **TUMOR LOCALIZATION** | **TREATMENT** | **TIME FREE FROM RECURRENCE (months)** | **RECURRENCE TREATMENT** | **STATUS** | **COMMENTS** |
| --- | --- | --- | --- | --- | --- | --- | --- | --- | --- | --- | --- | --- |
| Ref #55 | 1 | na (elderly) | M | 14 | fractures, pain | mesenchimal tumor (probably PMT) | Left ischium | surgery | 72 | na | cured | - |
| Ref #43 | 18 | na | na | na | na | na | na | na | na | na | na | - |
| Ref #56 | 11 | na | na | na | bone pain | PMT | foot/ankle and femur | surgery | 46,8 ± 2,3 | na | na | - |
| Ref #27 | 1 | na | F | 4 | bone pain, weakness, fractures, difficulty walking | PMT | L1 | surgery | 34 | surgery | cured | - |
| Ref #57 | 1 | 33 | F | na | na | giant cell granuloma-like | distal radius | surgery | 96 | na* | metastatic disease | *recurrence with metastasis to nasal cavity, lip, tongue and lungs |
| Ref #58 | 1 | 41 | F | 3 | bone pain, weakness, walk instability | PMT | right leg | surgery + radiotherapy | 12 | surgery | oral supplementation* | *pulmonary metastases (treatment not known) |
| Ref #59 | 1 | 34 | M | 24 | TIO | na | na | na | 288* | surgery | metastatic disease | * Primary tumor associated with TIO is not known. At the recurrence the patient presented a swelling in the right mandible (PMT) with lung metastasis, without TIO. |
| Ref #60 | 1 | 49 | M | 0.5 | muscle weakness and pain, cramps | PMT | left gastrocnemius and upper third of fibula | surgery | 1° recurrence: 12 2° recurrence: 120 | 1° recurrence: surgery 2° recurrence: surgery + long-acting octrotide analogue | oral supplementation | - |
| Ref #29 | 2 | 53 | F | 27 | bone pain, weakness, fractures | PMT | tibial plateau | curettage | 25 | surgery | cured | - |
|  |  | 55 | F | 8 | bone pain, weakness, fractures | PMT | tibial plateau | curettage | 8 | na | na | - |
| Ref #30 | 1 | 35 | F | 7 | back pain, fatigue, bone pain, muscle weakness | PMT | ilium | curettage and bone graft | na | surgery | cured | - |
| Ref #31 | 3 | 49 | F | 6 | TIO | PMT | nasal sinus | surgery | 8 | observation | oral supplementation | - |
|  |  | 52 | M | 7 | TIO | PMTMTC | knee | surgery | 12 | observation | oral supplementation | - |
|  |  | 53 | F | 27 | TIO | PMTMTC | tibia | surgery | 28 | observation | oral supplementation | - |
| Ref #61 | 1 | 34 | F | 6 | low back pain, weakness | plasmacytoma | T3 and T4 | surgery + radiotherapy | 60 | na | na | - |
| Ref #62 | 1 | 48 | M | 2 | fractures | PMTMTC | tongue | surgery | 1° recurrence: 12; 2° recurrence: 24 from the 1° surgery | 1° surgery; 2° radiotherapy | oral supplementation | - |
| Ref #63 | 1 | 45 | M | na | posterior neck pain, left hypoglossal nerve palsy, no evidence of osteomalacia; hypophosphatemia with normal alkaline phosphatase | PMTMTC | clivus region | surgery | 18 | stereotactic gamma knife radiosurgery + octreotide + oral supplementation | oral supplementation | The recurrence occurred with multiple bone fractures and bone pain: phosphatemia was not measured after 1° surgery |
| Ref #64 | 1 | 45 | F | 5 | bone pain, difficulty walking | PMTMTC | right distal fibula | surgery | 1° recurrence: 24; 2° recurrence: 60 from the 1° surgery; 3° recurrence: 72 from the 1° surgery; 4° recurrence: 120 from the 1° surgery*; 5° recurrence: 192 from the 1° surgery** | 1° recurrence: surgery 2° recurrence with metastasis to inguinal lymph nodes: surgery + radiotherapy 3° recurrence: surgery 4° recurrence: surgery + hemipelvectomy 5° recurrence: na | died | * tumor recurred and metastasized into the entire right lower extremity. **The tumor recurred in the ablated stump, and invaded the retroperitoneal space producing huge mineralized tumoral masses. The patient died of multiple abdominal involvement. |
| Ref #65 | 1 | 60 | F | na | right hip pain, osteomalacia, hypophosphatemia, change in behaviour | PMTMTC | right subfrontal mass | surgery | 36 | observation and oral supplementation with phosphate and vitamin D | oral supplementation | - |
| Ref #66 | 1 | 68 | F | 11 | hypophosphatemia, "osteoporosis" | PMT | meninges | surgery | 60* | conservative therapy due to patient conditions | oral supplementation** | * Recurrence presentation: fractures, bone pain ** patient's decision |
| Ref #34 | 2 | 73 | F | 13 | bone pain, weakness and difficulty in walking | haemangioperycitoma | ethmoid sinus | surgery | 48 | oral supplementation (no tumor detected) | died* | * died for colonic carcinoma |
|  |  | 43 | F | 7 | widespread muscoloskeletal pain | 1° cystic lymphangioma; 2° sclerosing haemangioma | left scapula | surgery | 36 | surgery + oral supplementation* | oral supplementation | *patient developed hypercalcaemia; nephrectomy due to pyonephrosis and renal calculus |
| Ref #67 | 1 | na | M | na | TIO | PMT with hemangiopericytoma-like vascular pattern | right maxillar sinus | surgery | 120 | surgery | cured | - |
| Ref #1 | 4 | 41 | F | 12 | TIO | PMTMTC | foot | surgery | 120 | surgery | cured | - |
|  |  | 53 | M | 20 | TIO | PMTMTC | leg and calf | surgery | 48 | surgery | cured | - |
|  |  | 32 | F | 4 | TIO | malignant PMTMCT | C1 | surgery | 24 | radiotherapy* | oral supplementation | * unresectable recurrence |
|  |  | 46 | M | 3 | TIO | haemangioperycitoma | ethmoid sinus | surgery | 12 | surgery | cured | - |
| Ref #68 | 2 | 58 | M | 20 | bone pain and fractures | PMTMTC | floor of mouth and mandible | surgery | 84 | surgery for multiple recurrence and lung metastasis with wedge resection | cured | - |
|  |  | 87 | F | nd | bone pain and fractures | PMTMTC | C2 | surgery (partial resection of tumor and C2 laminectomy) | na | na* | na | * multiple recurrence with normal chemistry |
| Ref #69 | 1 | 52 | F | 30 | osteomalacia, fractures | PMT | right distal femur + left ischial lesion abutting the acetabulum | curettage of both lesions | 9 | na (at the time of writing the patient was considering distal femoral resection and total knee arthroplasty) | na | - |
| Ref #70 | 1 | 44 | F | 1 | bone pain | chondroblastoma | right femur | surgery | 36 | surgery* + oral supplementation | metastatic disease | *pulmonary metastases |
| Ref #71 | 1 | 34 | F | 2 | bone pain | na | thyroid | surgery | 96 | surgery, oral supplementation | oral supplementation | - |

**Supplementary table 3:** demographics, clinical characteristics, localization procedures, tumor site and histology, treatments and outcome of patients with persistent TIO (Re), as retrieved from the literature, are shown (M=male; F=female; na=not available data; CHT=chemiotherapy).

|  | **N° of cases** | **AGE at DIAGNOSIS** | **SEX** | **CLINICAL PRESENTATION** | **DELAY in DIAGNOSIS** | **TUMOR LOCALIZATION** | **DIAGNOSTIC METHODS** | **TREATMENT** | **COMMENTS** |
| --- | --- | --- | --- | --- | --- | --- | --- | --- | --- |
| Ref #72 | 1 | 52 | F | general aches and pain, multiple vertebral fractures | na | SNC (not operable) | MRI, ^111^indium octreotide scintigraphy, ^68^Ga-DOTATATE PET/CT | Burosumab | - |
| Ref #26 | 8 | 38 | F | na | na | not localized | FDG-PET, MRI | oral supplementation | - |
|  |  | 52 | F | na | na | not localized | ^68^Ga-DOTATATE, CT | oral supplementation | - |
|  |  | 28 | F | na | na | not localized | ^68^Ga-DOTATATE, FDG-PET | oral supplementation | - |
|  |  | 31 | F | na | na | not localized | ^68^Ga-DOTATATE, CT | oral supplementation | - |
|  |  | 35 | F | na | na | not localized | FDG-PET, CT | oral supplementation | - |
|  |  | 19 | M | na | na | right obturator muscle* | ^68^Ga-DOTATATE, MRI | oral supplementation | *not operable |
|  |  | 28 | M | na | na | Head of left femur* | ^68^Ga-DOTATATE, MRI | oral supplementation | *patient's decision |
|  |  | 26 | F | na | na | Mid-left fibula* | ^68^Ga-DOTATATE, CT | oral supplementation | *patient's decision |
| Ref #14 | 11 | 50 | M | Weakness, fractures, ankle edema, muscle atrophy | na | not localized | Octreo-SPECT, FDG-PET/CT | na | - |
|  |  | 61 | M | Pain, fractures, osteomalacia on biopsy | na | not localized | Octreo-SPECT, FDG-PET/CT | na | - |
|  |  | 59 | M | Pain, weakness | na | not localized | Octreo-SPECT, FDG-PET/CT | na | - |
|  |  | 50 | M | Pain, muscle atrophy, weight loss, osteomalacia on biopsy | na | not localized | Octreo-SPECT, FDG-PET/CT | na | - |
|  |  | 50 | M | Pain, weakness, gait abnormality, low bone mineral density | na | not localized | Octreo-SPECT, FDG-PET/CT | na | - |
|  |  | 33 | M | Pain, weakness, fractures | na | not localized | Octreo-SPECT, FDG-PET/CT | na | - |
|  |  | 17 | M | Pain, fractures, low bone mineral density | na | not localized | Octreo-SPECT, FDG-PET/CT | na | - |
|  |  | 51 | F | Weakness, pain | na | not localized | Octreo-SPECT, FDG-PET/CT | na | - |
|  |  | 18 | F | Pain, weakness, myalgia, fractures, weight loss | na | not localized | Octreo-SPECT, FDG-PET/CT | na | - |
|  |  | 59 | M | Pain | na | not localized | Octreo-SPECT, FDG-PET/CT | na | - |
|  |  | 57 | M | Weakness, pain, falls, cramps, tetany | na | not localized | Octreo-SPECT, FDG-PET/CT | na | - |
| Ref #31 | 54 | na | na | na | na | na | na | na | patients with TIO without tumor localization (not further described) |
| Ref #34 | 1 | 61 | M | pain and weakness | 5 | not localized | ^99m^Tc-methylene diphosphonate scan, abdominal US, CT | oral supplementation | - |
| Ref #73 | 3 | 56 | F | pain and weakness | na | intravertebral localization* | na | oral supplementation | *not operable |
|  |  | 56 | F | pain and weakness | na | not localized | na | oral supplementation | - |
|  |  | 60 | M | pain and weakness | na | not localized | Octreotide Scan | oral supplementation | - |
| Ref #74 | 1 | 3 | M | limp, weakness, fractures | na | Jaw* | Radiographs, MRI | oral supplementation, beta-blockers, cinacalcet, RT | *not operable |
| Ref #75 | 1 | 20 | M | wrist pain | 10 | not localized | ^111^Indium-octreotide scan, FDG-PET/CT, MRI | oral supplementation | - |
